# Oral White Sponge Nevus: An Exceptional Differential Diagnosis in Childhood

**DOI:** 10.1155/2020/9296768

**Published:** 2020-08-26

**Authors:** Fatima zahra Elfatoiki, Sophia Capatas, Hayat Dahbi Skali, Fouzia Hali, Hicham Attar, Soumia Chiheb

**Affiliations:** Department of Dermatology, Ibn Rochd UHC, 1 Quartier des Hopitaux 20000, Casablanca, Morocco

## Abstract

White sponge nevus is an autosomal dominant skin disorder characterized by white, irregular, diffuse plaques mainly affecting the oral mucosa. Histological findings of white sponge nevus are characteristic but not pathognomonic. We report a case of an oral white sponge nevus in a 6-year-old girl, which poses a problem in differential diagnosis with oral candidiasis. No treatment was performed because of the benign and asymptomatic nature of the lesions.

## 1. Background

White sponge nevus is a rare hereditary mucosal disorder characterized by asymptomatic spongy white plaques that affect oral mucosa and less frequently nasal, esophageal, rectal, and genial mucosa. Oral white sponge nevus appears as white or gray diffuse plaques thickened with multiple furrows and spongy texture located onbuccal, labial, gingival mucosa and floor of the mouth [1].

## 2. Case Report

A 6-year-old girl without any parental consanguinity, presented to our Department of Dermatology with chronic white lesions of oral mucosa that appear at the age of 2 years according to her mother. She was treated as mucosal candidiasis for more than 3 years without any result. There were no other family members affected by similar lesions. The lesions were asymptomatic except of some episodic burning sensations when eating acid or spicy food.

**Figure 1:**
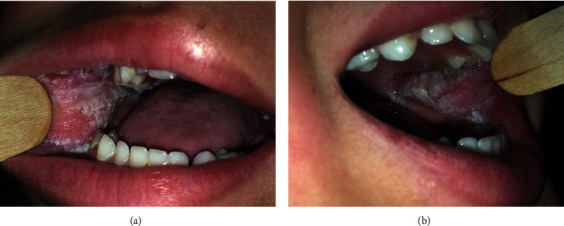
White irregular plaques with well-defined borders and symmetric distribution on the buccal mucosa.

Cutaneous examination showed white irregular plaques with well-defined borders and symmetric distribution on the buccal mucosa (Figure 1). There was no associated erythema, and the plaques did not scrape off when using a tongue blade. There were no similar lesions elsewhere on the other mucosae. Histopathological examination showed superficial parakeratosis, acanthosis, and spongiosis with perinuclear eosinophilic condensation of epithelial cells. A minimal lymphocytic infiltration was present in the stroma. PAS coloration was negative. These features were characteristic of oral white sponge nevus. The genetic study was not performed.

Because of the benign and asymptomatic nature of the lesions, no medication was performed and the fungal treatment was stopped.

## 3. Discussion

White sponge nevus is an autosomal dominant genodermatosis that is often manifested in early childhood and showed no gender preference [2]. In our case, the lesions appear at the age of 2 years and were treated as oral candidiasis for many years.

The autosomal dominant characteristic of white sponge nevus shows irregular penetrance and variable expressivity in the same family. In our case, no similar lesions were found in parents or in siblings. The mutations concern the Keratin 4 or Keratin 13 genes, encoding mucosa-specific keratin intermediate filament proteins Keratin 4 and Keratin 13, “respectively,” that are important for the assembly of keratin filaments [3, 4]. Liu et al. have recently compared sporadic and familial cases of white sponge nevus and concluded that only one of the five sporadic cases had keratin mutation [5]. The present case is probably sporadic without any similar familial cases especially in parents and siblings.

The lesions are seen usually in oral mucosa bilaterally as symmetrical, thickened, white, velvety, diffuse plaques that affect the buccal mucosa as observed in our case. The affected mucosa appears folded with a soft or spongy texture [6]. The less common intraoral location of white sponge nevus includes the tongue, labial mucosa, soft palate, alveolar mucosa, and floor of the mouth [7].

The differential diagnosis of oral white sponge nevus in childhood is made with other conditions presenting as white lesions on the oral mucosa [8]. These include other congenital disorders such as leukoedema, follicular keratosis, dyskeratosis congenita, hereditary benign intraepithelial dyskeratosis, and oral lesions of pachyonychia congenita and Darier disease. The acquired conditions include focal epithelial hyperplasia and oral florid papillomatosis associated with human papillomavirus. Oral candidiasis is difficult to differentiate from an oral white sponge nevus. This diagnosis is excluded by fungal examination and responsiveness to antifungal agents. Oral lichen planus may also be confused with an oral white sponge nevus, but this disorder is less common in young people [8].

The histological findings are characteristic of white sponge nevus. The histological features include acanthosis, hyperparakeratosis, and vacuolization of keratinocytes with a characteristic perinuclear eosinophilic condensation which is not necessarily pathognomonic [9].

Malignant transformation of a white sponge nevus is exceptional. Downham and Plezia reported the occurrence of an oral squamous-cell carcinoma within a white sponge nevus. This malignant transformation might be induced by chronic prednisone use, and no other similar cases were reported [10].

There is no treatment required in case of asymptomatic oral white sponge nevus. Various molecules were tested to reduce the clinical presentation of the white sponge nevus. These include beta-carotene, antibiotics (penicillin, azithromycin, etc.), antihistamines, local applications of retinoic acid, tetracycline mouth rinses, surgical resection, and laser ablation, but without any success [9].

In conclusion, our case illustrates the rarity of the oral white sponge nevus and its resemblance with other diagnoses more common in the oral cavity. The diagnosis of oral white sponge nevus should be established earlier by physicians to avoid unnecessary treatment.
